# Methyl 3,4,5-trimethoxycinnamate suppresses inflammation in RAW264.7 macrophages and blocks macrophage–adipocyte interaction

**DOI:** 10.1007/s10787-020-00720-8

**Published:** 2020-05-16

**Authors:** Olumayokun A. Olajide, Idowu S. Akande, Carlos da Silva Maia Bezerra Filho, Izabela Lepiarz-Raba, Damião Pergentino de Sousa

**Affiliations:** 1 Department of Pharmacy, School of Applied Sciences, University of Huddersfield, Huddersfield, HD1 3DH UK; 2 Department of Biochemistry, College of Medicine, University of Lagos, Lagos, Nigeria; 3 Laboratory of Pharmaceutical Chemistry, Federal University of Paraíba, João Pessoa, 58051-085 Brazil

**Keywords:** Methyl 3,4,5-trimethoxycinnamate, Anti-inflammatory, RAW264.7 macrophages, NF-κB, 3T3-L1 adipocytes, Natural product

## Abstract

Methyl 3,4,5-trimethoxycinnamate (MTC) is a bioactive natural phenylpropanoid. We evaluated anti-inflammatory effects of synthetic MTC in RAW264.7 macrophages and RAW264.7–3T3-L1 adipocytes co-culture. Levels of cytokines and chemokines, as well as NO and PGE_2_ in cell supernatants were analysed using ELISAs, Griess assay and enzyme immunoassays, respectively. In-cell cytoblot was used to assess levels of proteins; while DNA binding and reporter gene assays were used to measure transcription factor DNA binding and transcriptional activities, respectively. Glucose uptake in adipocytes was evaluated with 2‐deoxy‐2‐[(7‐nitro‐2, 1, 3‐benzoxadiazol‐4‐yl) amino]‐d‐glucose uptake. MTC (5–20 µM) suppressed LPS + IFNγ-induced release of TNFα, IL-6 and IL-1β, as well as NO/iNOS and PGE_2_/COX-2 levels in RAW264.7 cells. Furthermore, there was a reduction in phospho-IκB and phospho-p65 proteins, accompanied by a reduction in total IκB in RAW264.7 cells. Further studies showed that MTC also produced a reduction in NF-κB DNA binding and luciferase activity. Treatment of RAW264.7 cells with MTC (5–20 µM) resulted in enhanced DNA binding of Nrf2 and an increase in ARE-luciferase activity. In a macrophage–adipocyte co-culture, the compound reduced the release of TNFα, IL-6, IL-1β, MCP-1 and RANTES, while enhancing glucose uptake and activation of AMPKα. Our results suggest that MTC produced anti-inflammatory and antioxidant activities in macrophages. MTC also prevented inflammation in macrophage–adipocyte co-culture. The effect of MTC on glucose uptake in adipocytes is proposed to be linked to activation of AMPK.

**Electronic supplementary material:**

The online version of this article (10.1007/s10787-020-00720-8) contains supplementary material, which is available to authorized users.

## Introduction

Chronic inflammation still remains a fundamental hallmark of a wide variety of diseases, ranging from arthritis, asthma, inflammatory bowel disease, neurodegenerative disorders, cardiovascular diseases, cancer and metabolic disorders. Consequently, chronic inflammation and inflammatory signalling pathways remain prime targets in identifying new treatments for these inflammatory disorders.

Transcription factors such as the nuclear Factor kappa B (NF-κB) and nuclear factor erythroid 2-related factor (Nrf2) play critical roles in inflammation. NF-κB is responsible for the transcriptional control of pro-inflammatory genes, and plays a central role in inflammatory diseases such as rheumatoid arthritis, inflammatory bowel disease, and autoimmunity, as well as diseases comprising a significant inflammatory component such as cancer and atherosclerosis (Mitchell and Carmody 2018). On the other hand, Nrf2 modulates constitutive or inducible molecular systems regulating redox homeostasis, resulting in the activation of antioxidants, anti-inflammatory molecules, as well as phase I and II drug metabolising enzymes (Sivandzade et al. 2019). Nrf2 has been functionally linked to cytoprotection in low-grade stress, chronic inflammation, metabolic alterations, and reactive oxygen species formation (Cuadrado et al. 2018). Interestingly, studies have established that there is a cross-talk between Nrf2 and NF-κB signalling pathways; the absence of Nrf2 enhances NF-κB activity with the resulting increase in pro-inflammatory cytokine production (Wardyn et al. 2015). Furthermore, studies have shown that Nrf2 is able to negate the transcriptional up-regulation of pro-inflammatory genes through mechanisms that are independent of its redox regulatory activity (Kobayashi et al. 2016).

Accumulating evidence suggests that metabolic conditions induce chronic low-grade macrophage-mediated inflammation in colon, liver, muscle and adipose tissue (Li et al. 2018). In this context, pro-inflammatory cytokines are secreted by recruited and resident macrophages in these organs and play a significant role in linking inflammation and insulin signalling in adjacent metabolic cells (Shapiro et al. 2011). Furthermore, it is widely known that obesity, insulin resistance and type two diabetes are closely linked with chronic inflammation, which is accompanied by excessive production of pro-inflammatory cytokines, acute-phase reactants, and activation of inflammatory signalling pathways (Wellen and Hotamisligil 2005; Hotamisligil 2006). Consequently, an important strategy in targeting insulin resistance will involve the use of anti-inflammatory modalities.

Anti-inflammatory natural compounds are now considered to possess significant therapeutic potentials in metabolic syndrome/insulin resistance. Flavonoids are plant-based anti-inflammatory natural products which have been suggested to been suggested to reduce the risk of diabetes by targeting inflammatory signals (Ren et al. 2019).

**Fig. 1:**
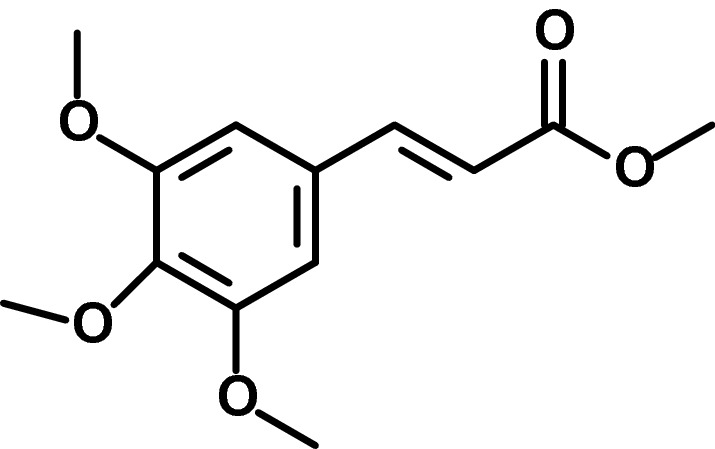
Chemical structure of Methyl 3,4,5-trimethoxycinnamate

Methyl 3,4,5-trimethoxycinnamate (Fig. 1) is a natural phenylpropanoid ester found in several plants and has anti-platelet aggregation (Tsai et al. 2005) and antiarrhythmic activities (Zhao et al. 2013). The precursor of this compound 3, 4, 5-trimethoxycinnamic acid, is found in nature and has various pharmacological activities, such as anti-stress (Kawashima et al. 2004) and immunocompetent actions (Yu et al. 2014). Reports have shown that phenylpropanoids isolated from *Polygala tenuifolia* inhibited lipopolysaccharide (LPS)-induced nitric oxide production in BV-2 microglia (Cho et al. 2012). In the present study, methyl 3,4,5-trimethoxycinnamate (MTC) was prepared via fischer esterification using 3,4,5-trimethoxycinnamic acid as the starting material, as previously reported (da Nóbrega et al. 2018). We then investigated the compound for effects on inflammation and Nrf2 antioxidant mechanisms in RAW264.7 macrophages. We also determined effects of MTC on inflammation in a macrophage–adipocyte co-culture stimulated with LPS and gamma interferon (IFNγ).

## Materials and methods

### Synthesis of methyl 3,4,5-trimethoxycinnamate

MTC was synthesised as earlier described (da Nóbrega et al. 2018). To a solution of 3,4,5-trimethoxycinnamic acid (0.25 g) in 250 mL of MeOH, 0.5 mL 96% (v/v) H_2_SO_4_ was added under stirring. The reaction mixture was refluxed for 3 h. Half of MeOH was removed under reduced pressure and the following solution was then diluted with 10-mL water and the product extracted with ethyl acetate. The organic phase of the reaction was successively washed with 5% (w/v) NaHCO_3_ (twice) and water, dried over anhydrous Na_2_SO_4_ and filtered. After removal of ethyl acetate under vacuum, the methyl 3,4,5-trimethoxycinnamate was obtained.

### Cell culture

RAW264.7 mouse macrophages (ECACC 91062702) were cultured in Dulbecco’s modified eagle’s medium (DMEM), supplemented with 2-mM glutamine and 10% foetal bovine serum (FBS). Cell viability experiments were carried out to determine whether incubation of RAW264.7 macrophages with concentrations of MTC used in combination with LPS (1 µg/mL) and IFNγ (10 ng/mL) would produce cytotoxic effects (Supplementary data 1).

3T3-L1 (ATCC^®^ CL-173™) mouse pre-adipocytes were cultured in DMEM and supplemented with 2-mM glutamine and 10% FBS. All cells were cultured in an incubator at 37 °C and 5% CO_2_.

### Determination of pro- and anti-inflammatory mediators in culture supernatants of LPS + IFNγ-stimulated RAW264.7 cells

Cultured RAW264.7 macrophages were treated with MTC (5–20 µM). Thirty minutes later, the cells were stimulated with LPS (1 µg/mL) and IFNγ (10 ng/mL) for a further 24 h. Culture supernatants were analysed for levels of NO using the Griess assay kit (Promega), according to the manufacturer’s instructions. Levels of pro-inflammatory (TNFα, IL-6 and IL-1β) and anti-inflammatory (IL-10) cytokines in culture supernatants were measured using mouse ELISA kits (Biolegend), according to the manufacturer’s instructions. PGE_2_ production was measured using PGE_2_ EIA assay kit (Arbor Assays), according to the manufacturer’s instructions.

### Luciferase reporter gene assays

RAW264.7 cells were seeded out in a 24-well plate (2 × 10^5^ cells per well) in Opti-MEM (Gibco) and incubated at 37 °C for 4 h. Cells were then transfected with either the pNL3.2.NF-κB-RE [NlucP/NF-κB-RE/Hygro] vector (Promega, UK) or the pGL4.37[luc2P/ARE/Hygro] vector (Promega, UK) using lipofectamine 2000 transfection reagent (Invitrogen) and incubated for a further 4 h. Thereafter, experiments were carried out to evaluate the effects of MTC (5–20 µM) on NF-κB and antioxidant response elements (ARE) luciferase activities, respectively. Luciferase activities were measured using Dual-Glo luciferase assay kit (Promega, Southampton, UK) according to the manufacturer’s instructions.

### NF-κB and Nrf2 DNA binding assays

DNA binding assays were carried out to evaluate the effects of MTC on DNA binding of the NF-κB transcription factor in LPS + IFNγ-stimulated RAW264.7 macrophages. These assays were also used to assess the ability of MTC to induce the binding of Nrf2 transcription factor to ARE consensus sites in the DNA. Cultured RAW264.7 cells were treated with MTC (5–20 µM) for 30 min, followed by stimulation with LPS (1 µg/mL) and IFNγ (10 ng/mL) for a further 24 h in experiments to determine DNA binding of NF-κB. The cells were also treated with MTC (5–20 µM) for 24 h to evaluate effects on binding of Nrf2 to ARE consensus sites. Nuclear extracts were collected and analysed for binding of transcription factors to the DNA using the TransAM NF-κB and Nrf2 transcription factor EMSA kits (Activ Motif, Belgium) according to the manufacturer’s instructions. The TransAM transcription factor assay kits employed a 96-well plate to which oligonucleotides containing the NF-κB consensus site (5′-GGGACTTTCC-3′) or the ARE consensus binding site (5′-GTCACAGTGACTCAGCAGAATCTG-3′) have been immobilised.

### In-cell western/cytoblot

The in-cell western is now an established method for the rapid quantification of proteins in cells (Velagapudi et al. 2019b), because it combines the specificity of western blotting with the quantification capability of ELISA. In these experiments, RAW264.7 macrophages were seeded into a 96-well plate (5 × 10^4^ cells/mL). At 70% confluence, cells were treated with MTC (5–20 μM) for 30 min, followed by stimulation with LPS (1 µg/mL) and IFNγ (10 ng/mL) for different incubation periods. Cells were fixed with 8% paraformaldehyde solution (100 μL) for 15 min. and then washed with PBS. The cells were then incubated with the primary antibodies overnight at 4 °C. The following antibodies were used: rabbit anti-COX-2 (Abcam), rabbit anti-iNOS (Abcam), rabbit anti-phospho-p65 (Cell Signalling technologies), rabbit anti-phospho-IκB (Santa Cruz Biotechnology), rabbit anti-IκB (Santa Cruz Biotechnology), and rabbit anti-phospho-AMPKα (Santa Cruz Biotechnology). Thereafter, cells were washed with PBS and incubated with anti-rabbit HRP secondary antibody for 2 h at room temperature. Then, 100-µL HRP substrate was added to the plate and signal measured at 450 nm with a microplate reader. Readings were normalised with Janus Green stain (Abcam).

### Differentiation of 3T3-L1 pre-adipocytes

Sub-confluent 3T3-L1 pre-adipocytes were differentiated into adipocytes using differentiation medium (DMEM) containing 10% FBS supplemented with 0.5-mm 3-isobutyl-1-methylxanthine (0.5 mM), dexamethasone (1 µM) and insulin (200 nM). Adipocyte differentiation was assessed using the oil red O staining quantitative assay kit (Cayman). Culture supernatants were removed from cells, followed by washing with PBS. Thereafter, lipid droplets assay fixative was added to each well and incubated for 15 min at room temperature. Following removal of the fixative, cells were washed and 75-μL oil red O solution was added to each well for 20 min at room temperature. This was followed by removal of the oil red O and thorough washing of the wells with 100-μL wash solution and the addition of 100-μL dye extraction solution and absorbance read on a Tecan F50 microplate at 492 nm. Differentiation was quantitatively confirmed by comparing the absorbance of differentiated and undifferentiated cells (Supplementary data 2).

### RAW264.7-differentiated 3T3-L1 adipocytes co-culture

Following differentiation, 3T3-L1 adipocytes were co-cultured with RAW264.7 macrophages using the Transwell system (0.4 µm porous membrane; Corning). In the co-culture, differentiated 3T3-L1 cells (1 × 10^5^) were cultured in the lower well; while, 5 × 10^4^ RAW264.7 macrophages were cultured in inserts that constituted the upper chamber. Following the establishment of co-culture, RAW264.7 cells in the upper chamber were treated with MTC (5–20 µM) for 30 min, followed by stimulation with LPS (1 µg/mL) and IFNγ (10 ng/mL) for a further 24 h. Culture supernatants were collected and analysed for levels of NO, TNFα, IL-1β, IL-6 using similar protocols described in experiments on RAW264.7 macrophage mono-culture. Production of monocyte chemoattractant protein-1 (MCP-1/CCL2) was measured using a mouse CCL2 ELISA kit (Invitrogen); while, levels of RANTES in culture supernatants were detected with a mouse CCL5/RANTES ELISA kit (R and D Systems).

Glucose uptake was determined by incubating differentiated 3T3-L1 adipocytes in the lower chamber with 2‐deoxy‐2‐[(7‐nitro‐2, 1, 3‐benzoxadiazol‐4‐yl) amino]‐d‐glucose (2‐NBDG) (100 µM) in glucose-free DMEM for a further 1 h after treatment. Thereafter, cells were washed with PBS and glucose uptake by fluorescence detection at an excitation/emission wavelength of 485 nm/535 nm.

### Statistical analyses

Values of all experiments were represented as a mean ± SEM of at least three experiments. Values were compared using one-way ANOVA followed by a post hoc Tukey test. In experiments to confirm differentiation of adipocytes, differences in oil red O staining between undifferentiated and differentiated adipocytes were determined using the Student’s *t* test.

## Results

### Structural characterisation of methyl 3,4,5-trimethoxycinnamate

Yield 93%; white solid; 1H NMR (200 MHz, CDCl_3_) δ_H_ 7.59 (d, *J* = 16.0 Hz, 1H), 6.73 (s, 2H), 6.33 (d, *J* = 16.0 Hz, 1H), 3.87 (s, 9H), 3.79 (s, 3H). ^13^C NMR (50 MHz, CDCl_3_) δ_C_ 167.5, 153.5, 144.9, 140.1, 129.9, 117.1, 105.2, 61.1, 56.2, 51.8, IR ѵ_max_ (KBr, cm^−1^) 3003, 1697, 2943, 2837, 1632, 1040, 1005, 1582, 1506, 851.

### MTC produced anti-inflammatory activity in LPS + IFNγ-stimulated RAW264.7 macrophages

**Fig. 2:**
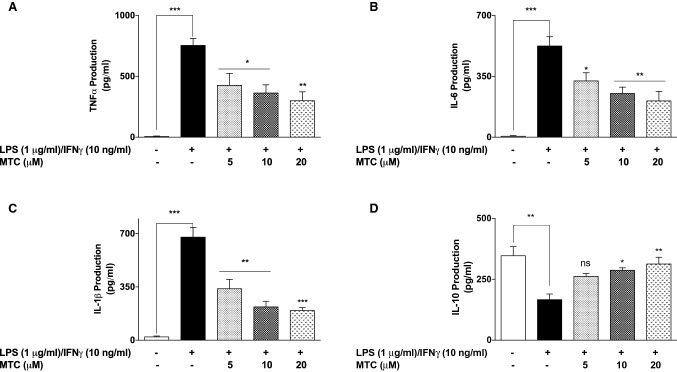
Pre-treatment of RAW264.7 macrophages with MTC (5–20 µM) prior to stimulation with LPS (1 µg/mL) and IFN (10 ng/mL) for 24 h resulted in reduction in the concentrations of TNFα (**a**), IL-6 (**b**), and IL-1β (**c**). Levels of IL-10 were increased, compared with LPS stimulation (**d**). Results are mean ± SEM of three independent experiments. Data were analysed using one-way ANOVA for multiple comparisons with post–hoc Tukey test. *p < 0.05, **p < 0.01, ***p < 0.001, *ns* not significant, compared with LPS + IFNγ

In experiments to determine whether MTC produces anti-inflammatory activity, RAW265.7 cells were stimulated with IFNγ (10 ng/mL) and LPS (1 µg/mL) for 24 h. This resulted in ~ 80-fold increase in TNFα secretion into the culture supernatant in comparison with control cells. However, when cells were treated with 5-, 10- and 20-µM MTC, there was ~ 1.8-, 2.1- and 2.5-fold reduction in LPS + IFNγ-induced increase in TNFα production, respectively (Fig. 2a). MTC (5–20 µM) produced similar effects on the release of IL-6 and IL-1β in LPS + IFNγ-stimulated RAW264.7 cells (Fig. 2b, c).

In other experiments, LPS + IFNγ stimulation caused a significant (*p* < 0.01) reduction in the levels of IL-10 in RAW264.7 cells (Fig. 2d). On pre-treating the cells with MTC (5 µM), there was a statistically insignificant increase in IL-10 release. However, when the concentrations of MTC were increased to 10 and 20 µM, significant (*p* < 0.05) increase in IL-10 levels was observed in culture supernatants.

**Fig. 3:**
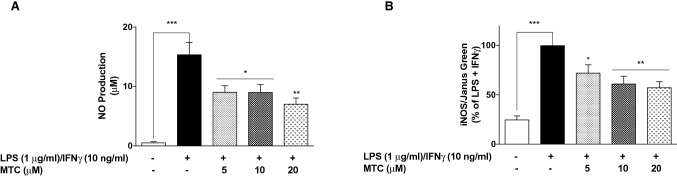
MTC (5–20 µM) reduced NO production **a** and iNOS protein levels **b** in RAW264.7 macrophages stimulated with LPS (1 µg/mL) and IFN (10 ng/mL) for 24 h. Results are mean ± SEM of three independent experiments. Data were analysed using one-way ANOVA for multiple comparisons with post–hoc Tukey test. *p < 0.05, **p < 0.01, ***p < 0.001, compared with LPS + IFNγ

**Fig. 4:**
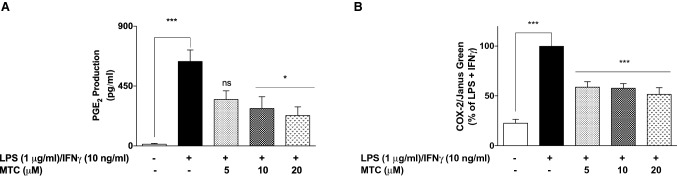
MTC (5–20 µM) reduced PGE_2_ production **a** and COX-2 protein levels **b** in RAW264.7 macrophages stimulated with LPS (1 µg/mL) and IFN (10 ng/mL) for 24 h. Results are mean ± SEM of three independent experiments. Data were analysed using one-way ANOVA for multiple comparisons with post–hoc Tukey test. *p < 0.05, ***p < 0.001, *ns* not significant, compared with LPS + IFNγ

To further confirm the anti-inflammatory activity of MTC, we measured the levels of NO (in form of nitrite) released in culture supernatants obtained from LPS + IFNγ-stimulated cells, and observed that pre-treatment with MTC (5–20 µM) resulted in significant (*p* < 0.05) reduction in NO production. The results show that NO concentrations in cells stimulated with LPS + IFNγ alone was ~ 15.4 µM. This was reduced to ~ 9.1, ~ 9.0, and ~ 7.1 µM in cells pre-treated with 5, 10 and 20 µM of MTC, respectively (Fig. 3a). We also observed that LPS + IFNγ-induced increase in iNOS protein levels were markedly reduced in the presence of MTC (5–20 µM) (Fig. 3b). It was observed that increases in PGE_2_ production as well as elevation in COX-2 protein expression caused by stimulating RAW264.7 cells with LPS + IFNγ were significantly reduced in the presence of 10 and 20 µM of MTC. Reductions observed with 5 µM of the compound were not significant (Fig. 4a, b).

### Anti-inflammatory activity of MTC is mediated through inhibition of NF-κB activity

**Fig. 5:**
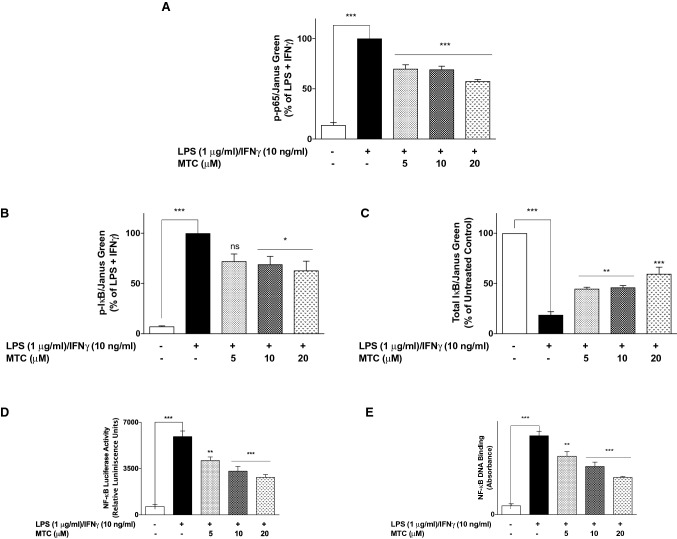
**a** MTC (5–20 µM) reduced phosphorylation of p65 in the cytoplasm of LPS (1 µg/mL) + IFNγ (10 ng/mL)-stimulated RAW264.7 macrophages following 60-h incubation. **b** Reduction in the levels of phospho-IκBα in LPS (1 µg/mL) + IFNγ (10 ng/mL)-stimulated RAW264.7 macrophages by MTC (5–20 µM). **c** MTC (5–20 µM) prevented LPS (1 µg/mL) + IFNγ (10 ng/mL)-induced degradation of IκBα in RAW264.7 macrophages. MTC inhibited NF-κB-mediated gene transcription (**d**) and DNA binding in LPS (1 µg/mL) + IFNγ (10 ng/mL)-stimulated RAW264.7 macrophages (**e**). Results are mean ± SEM of three independent experiments. Data were analysed using one-way ANOVA for multiple comparisons with post–hoc Tukey test. *p < 0.05, ***p < 0.001, *ns* not significant, compared with LPS + IFNγ

Based on results showing anti-inflammatory activity of MTC, we were next interested to determine whether the compound produced this action by targeting NF-κB signalling in RAW264.7 macrophages. Results of in-cell western blotting in Fig. 5a–c show that stimulating RAW264.7 cells resulted in marked increase in phosphorylation of p65 sub-unit and IκB protein, accompanied by degradation of IκB protein. Pre-treatment of RAW264.7 cells with MTC prior to stimulation with LPS + IFNγ resulted in significant (*p* < 0.001) reduction in the expression of phospho-p65 protein at all the concentrations investigated (Fig. 5a). Interestingly, at 5-µM concentration of MTC, reduction in the levels of phospho-IκB was not significant. However, at higher concentrations of 10- and 20-µM, phosphorylation of IκB was significantly (*p* < 0.05) reduced (Fig. 5b). Furthermore, LPS + IFNγ-induced degradation of IκB was significantly (*p* < 0.01) reduced by MTC (5–20 µM) (Fig. 5c).

In reporter gene assay experiments, LPS + IFNγ stimulation of RAW264.7 cells which were transiently transfected with the luciferase-driven NF-κB reporter resulted in a significant (*p* < 0.001) increase in luciferase activity (Fig. 5d). Treatment of the cells with MTC (5–20 µM) prior to inflammatory stimulation resulted in significant (*p* < 0.01) and concentration-dependent reduction in luciferase activity. Similarly, it was observed that LPS + IFNγ-induced DNA binding of NF-κB was significantly (*p* < 0.01) reduced in a concentration-dependent fashion (Fig. 5e).

### MTC activated Nrf2/HO-1 protective mechanism in RAW264.7 macrophages

**Fig. 6:**
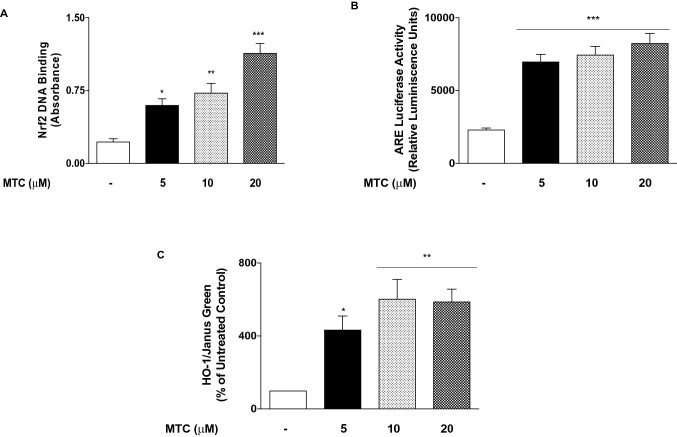
Activation of Nrf2/HO-1 by MTC in RAW264.7 nacrophages. MTC (5–20 µM) treatment increased DNA binding of Nrf2 to immobilised ARE consensus binding site in BV-2 microglia (**a**). MTC activated ARE-luciferase activity in RAW264.7 macrophages transfected with ARE construct (**b**). Increase in HO-1 protein levels by treatment with MTC (5–20 µM). Results are mean ± SEM of three independent experiments. Data were analysed using one-way ANOVA for multiple comparisons with post–hoc Tukey test. *p < 0.05, ***p < 0.001, *ns* not significant, compared with untreated control

We also investigated whether MTC produces antioxidant effects in RAW264.7 cells through activation of Nrf2 and the subsequent increase in the expression of HO-1 protein. We observed that the degree of DNA binding of Nrf2 increased ~ threefold from baseline (control) values when the cells were treated with 5-µM MTC. However, binding increased ~ 3.2- and ~ 5.2-fold with 10- and 20-µM concentrations of MTC, respectively (Fig. 6a). To further establish the effect of MTC on Nrf2 activity, we carried out a reporter gene assay to determine whether the compound would have an effect on luciferase-driven ARE activity. Results in Fig. 6b show that treatment with MTC (5–20 µM) resulted in significant (*p* < 0.001) increase in ARE-luciferase activity, when compared with untreated control cells. Experiments to evaluate the effect of MTC on HO-1 protein showed that treatment of RAW264.7 cells with MTC (5–20 µM) caused a significant (*p* < 0.05) increase in protein levels of HO-1 in the cells (Fig. 6c).

### Treatment with MTC prevents inflammation in RAW264.7–3T3-L1 adipocytes co-culture

**Fig. 7:**
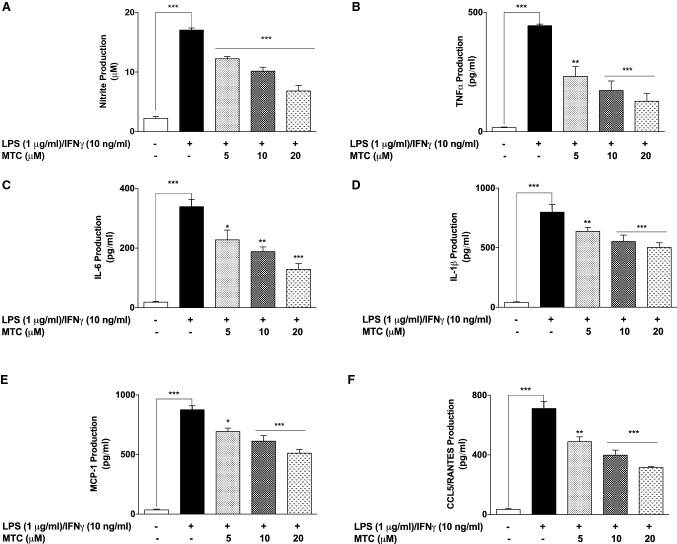
Pre-treatment of RAW264.7 macrophage–3T3-L1 adipocyte co-culture with MTC (5–20 µM) prior to stimulation with LPS (1 µg/mL) and IFN (10 ng/mL) for 24 h resulted in reduction in the concentrations of NO (**a**), TNFα (**b**), IL-6 (**c**), IL-1β (**d**), MCP-1 (**e**) and CCL5/RANTES (**f**). Results are mean ± SEM of three independent experiments. Data were analysed using one-way ANOVA for multiple comparisons with post–hoc Tukey test. *p < 0.05, **p < 0.01, ***p < 0.001, compared with LPS + IFNγ

Results in Fig. 7a show that addition of LPS (1 µg/mL) and IFNγ (10 ng/mL) to the RAW264.7 layer of macrophage–adipocyte co-culture resulted in an increase in the concentration of NO in the culture supernatants. However, treatment with MTC (5–20 µM) prior to inflammatory stimulation resulted in a significant (*p* < 0.001) and concentration-dependent reduction in NO production. Similar observations were made following analyses of culture supernatants for levels of pro-inflammatory cytokines TNFα (Fig. 7b), IL-6 (Fig. 7c) and IL-1β (Fig. 7d).

To evaluate the effects of MTC on the secretion of chemokines implicated in inflammation-mediated insulin resistance, we analysed culture supernatants and found that following stimulation of the RAW264.7 layer with LPS (1 µg/mL) and IFNγ (10 ng/mL), there was ~ 23-fold increase in the levels of MCP-1, in comparison with control cells. Supernatants from cells that were pre-treated with 5, 10 and 20 μM of MTC showed ~ 1.30-, ~ 1.42- and ~ 1.71-fold reduction in the concentrations of MCP-1, respectively (Fig. 7e). Similarly, inflammatory stimulation of RAW264.7 layer caused a significant (*p* < 0.001) elevation in the levels of RANTES in the culture supernatants, and these were significantly (*p* < 0.01) reduced following pre-treatment with MTC (5–20 μM) (Fig. 7f).

### MTC enhances glucose uptake through activation of AMPKα in 3T3-L1 adipocytes

**Fig. 8:**
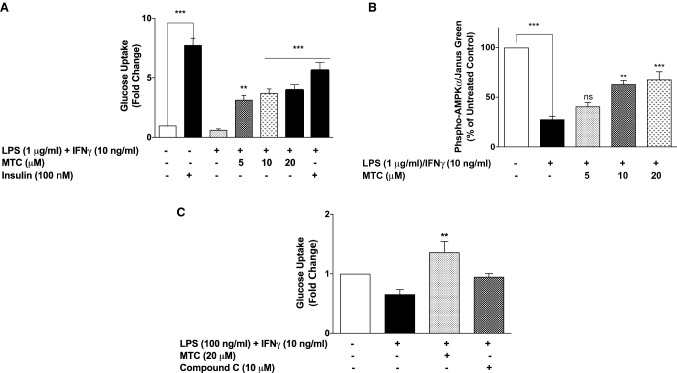
Treatment of macrophage–adipocyte co-culture with MTC (5–20 µM) prior to LPS (1 µg/mL) and IFN (10 ng/mL) enhanced 2‐NBDG uptake in adipocytes (**a**), and activated AMPKα (**b**). The enhancement of 2‐NBDG uptake by MTC (20 µM) was partially prevented in the presence of the AMPK inhibitor, compound C (**c**). Results are mean ± SEM of three independent experiments. Data were analysed using one-way ANOVA for multiple comparisons with post hoc Tukey test. *p < 0.05, **p < 0.01, ***p < 0.001, *ns* not significant

Based on our observation on the effects of MTC on inflammation-induced MCP-1 and RANTES secretion in a co-culture of RAW264.7 macrophages and 3T3-L1 adipocytes, we became interested in evaluating whether these effects were accompanied by enhancement of glucose uptake in the adipocytes. Expectedly, treatment of adipocytes with only insulin (100 nM) for 24 h caused a marked increase in 2‐NBDG uptake by the cells, in comparison with untreated cells (Fig. 8a). However, we also observed that stimulation of the macrophage layer of the co-culture with LPS (1 µg/mL) and IFNγ (10 ng/mL) for the same period of time resulted in a slight reduction in 2‐NBDG uptake when compared with control. Interestingly, treatment with MTC (5–20 μM) prior to inflammatory stimulation of RAW264.7 macrophages resulted in significant (*p* < 0.01) increase in 2‐NBDG uptake (Fig. 8a).

Stimulation of the macrophage layer with LPS (1 µg/mL) and IFNγ (10 ng/mL) caused a significant (*p* < 0.001) reduction in phospho-AMPKα protein in adipocytes after 24 h (Fig. 8b). Pre-treatment with MTC (5 μM) did not have significant effect on AMPKα activation. However, when the concentration of the compound was increased to 10 and 20 μM, there was a significant inhibition of deactivation of adipocyte AMPKα induced by inflammatory stimulation of macrophages (Fig. 8b).

In Fig. 8c we show that in the presence of the AMPKα inhibitor, compound C (10 μM), increase in adipocyte glucose uptake following treatment with MTC (20 μM) was insignificantly (*p* < 0.05) reversed.

## Discussion

Macrophage-mediated chronic inflammation is known to be a major contributor in many diseases, and several strategies are currently being deployed in preventing or blocking inflammation. Secondary metabolites from natural products have been extensively investigated as a potential source of new chemical scaffolds for new anti-inflammatory drugs. In this study, we have shown that a synthetic form of natural 3, 4, 5-trimethoxycinnamic acid, MTC reduced the release of pro-inflammatory cytokines TNFα, IL-6 and IL-1β in LPS + IFNγ-stimulated RAW264.7 cells, while increasing the production of the anti-inflammatory cytokine IL-10. We further observed that MTC treatment resulted in the reduction of both NO and PGE_2_ release through mechanisms involving inhibition of iNOS and COX-2 proteins, respectively. These effects demonstrate anti-inflammatory activity of MTC.

The release of pro-inflammatory mediators from macrophages has been implicated in many inflammatory and auto-immune diseases. In rheumatoid arthritis, macrophages secrete potent cytokines and chemokines including TNF, IL-1, IL-6, IL-10, IL-12, IL18, IL-15, IL-10, GM-CSF, M-CSF and TGFβ which contribute to the chronic inflammation associated with the disease (Siouti and Andreakos 2019). Pro-inflammatory macrophages have also been linked to diseases such as atherosclerosis (Bäck et al. 2019), inflammatory bowel diseases (Heinsbroek and Gordon 2009) and the metabolic syndrome (Kumar 2019). The anti-inflammatory activity of MTC, therefore, suggests a potential therapeutic strategy in treating some of these conditions.

Studies have suggested that phenylpropanoids show anti-inflammatory activity in a number of experimental models. Phenylpropanoids isolated from the Chinese olive (*Canarium album*) and *Polygala tenuifolia* were reported to inhibit neuroinflammation in LPS-activated BV-2 microglia (Cho et al. 2012; Zhang et al. 2019). Similarly, phenylpropanoids isolated from *Ficus hirta* and *Brassica oleracea* inhibited LPS*-*induced nitric oxide production in RAW 264.7 macrophages (Lee et al. 2014; Cheng et al. 2017). Interestingly, some synthetic derivatives of 3, 4, 5-trimethoxycinnamic acid have been suggested to have anti-inflammatory activity in TNFα-stimulated endothelial cells (Kumar et al. 2011). To our knowledge, this is the first study demonstrating anti-inflammatory activity of MTC in murine macrophages, and suggests that this compound is a potential chemical scaffold for the identification of novel anti-inflammatory agents.

The transcription factor NF-κB is now widely accepted as the master regulator of the genes encoding inflammatory proteins, including iNOS, COX-2, TNFα, IL-6 and IL-1β. NF-κB is also an important target for the discovery of new drugs for treating for various inflammatory disorders. Based on our results showing that MTC produces anti-inflammatory activity in RAW264.7 macrophages, we carried out further investigations to determine whether this compound could target the activation of NF-κB. Our experiments revealed that MTC suppressed IκB-mediated activation and nuclear DNA binding of NF-κB, as well as NF-κB-mediated gene transcription in RAW264.7 cells. The effects of the compound on NF-κB further confirms its anti-inflammatory activity in peripheral macrophages.

Investigations by us and others have suggested that the anti-inflammatory action of some compounds could be due, at least in part, to their ability to activate the Nrf2 transcriptions factor (Onasanwo et al. 2016; Okorji et al. 2016; Velagapudi et al. 2017; 2019a; Zhou et al. 2018). This prompted us to explore and show that MTC increased DNA binding of Nrf2 and enhanced ARE-mediated antioxidant gene transcription in RAW264.7 cells. While this is the first evidence linking MTC to activation of Nrf2, studies reported by Schadich et al. (2016) suggest that ginger (*Zingiber officinale*) phenylpropanoids activate Nrf2 in human keratinocytes. Our studies could not establish a link between the anti-inflammatory activity and activation of Nrf2/ARE mechanisms by MTC, thus warranting further investigations.

Studies have shown pro-inflammatory cytokines secreted by recruited and resident macrophages in adipocytes play a significant role in linking inflammation and insulin signalling (Shapiro et al. 2011). Specifically, pro-inflammatory cytokines like TNFα, which are secreted as a result of the interaction between adipocytes and macrophages have been linked to dysfunctions in glucose metabolism and insulin resistance (Guilherme et al. 2008). Consequently, we investigated effects of MTC on inflammatory responses in a co-culture of RAW264.7 macrophages and differentiated 3T3-L1 adipocytes. Our results revealed that stimulating the macrophage layer of the co-culture resulted in significant increases in the concentrations of NO as well as the pro-inflammatory cytokines TNFα, IL-6 and IL-1β. These observations reflect earlier reports linking significant production of pro-inflammatory cytokines following LPS stimulation of macrophage–adipocyte interactions (Yamashita et al. 2007; Fite et al. 2015; Engin 2017; Gothai et al. 2016). Results also showed that MTC prevented macrophage-mediated inflammation in adipocytes, through reduction of elevated levels of adipocyte-specific chemokines MCP-1 and RANTES following inflammatory stimulation of adjacent macrophages. Emerging evidence from several reports have suggested that the use of anti-inflammatory phytochemicals such as flavonoids could be a viable strategy in treating insulin resistance (Miranda et al. 2015; Gun and Lambert 2013; Merone and McDermott 2017). Similar properties have been reported for some novel multi-targeting anti-inflammatory glitazones (Elzahhar et al. 2019). The potential effect of MTC in preventing inflammation-mediated insulin resistance was further confirmed in experiments demonstrating its ability to enhance glucose uptake in adipocytes.

AMPK is an energy sensor that controls glucose metabolism, and has been suggested to suppress the pro-inflammatory environment in adipocytes (Bijland et al. 2013). We showed in this study that MTC suppressed macrophage activation-mediated dephosphorylation of AMPKα in adipocytes, suggesting that this kinase plays a role in the effects of the compound in reducing inflammation and enhancing glucose uptake. This was further confirmed in pharmacological antagonism experiments with the AMPK inhibitor compound C, which show that the glucose uptake promoting effect of MTC in adipocytes was partially reversed.

This study has established that MTC produced anti-inflammatory activity in both RAW264.7 macrophages and in a macrophage–adipocyte co-culture. It is further suggested that the anti-inflammatory activity of this compound possibly contributes to its ability to enhance glucose uptake in adipocytes through mechanisms involving activation of AMPK. It is not clear if this compound is able to affect the levels of other markers of inflammation and insulin resistance in adipocytes; further studies are establishing these possible effects.

## Electronic supplementary material

Below is the link to the electronic supplementary material.

Supplementary material 1 (PPTX 114 kb)
