# Supplementary figures and images for: Methyl 3,4,5-trimethoxycinnamate suppresses inflammation in RAW264.7 macrophages and blocks macrophage–adipocyte interaction
Source: Inflammopharmacology. 2020 May 16;28(5):1315–26. doi: 10.1007/s10787-020-00720-8 (PMC7524821; doi:10.1007/s10787-020-00720-8)

## Slide 1
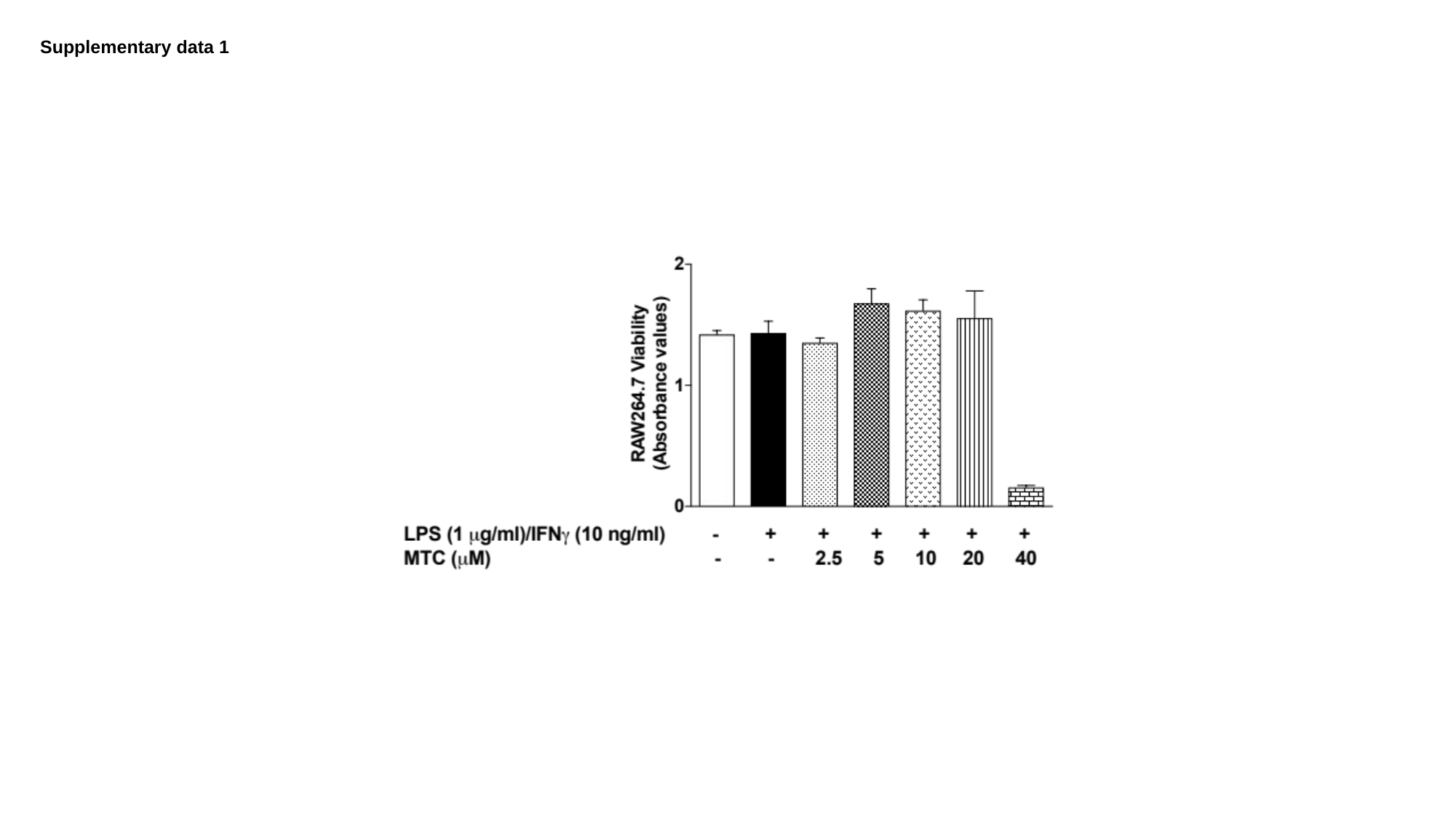

Supplementary data 1

## Slide 2
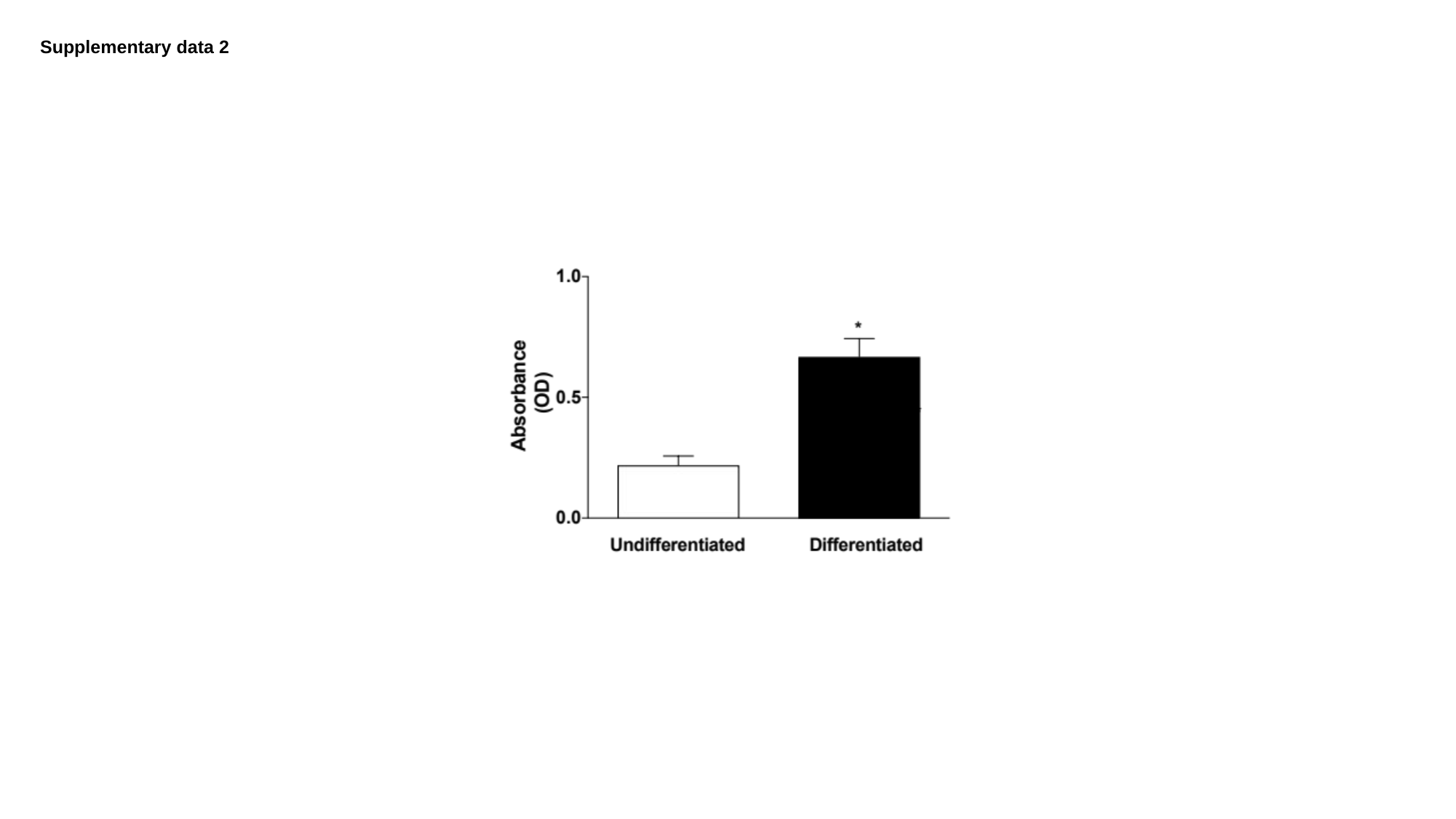

Supplementary data 2

Supplement: Supplementary file 1 — Supplementary material 1 (PPTX 114 kb) [file 10787_2020_720_MOESM1_ESM.pptx]
